# Acyclovir-induced neurotoxicity with a positive cerebrospinal fluid varicella zoster PCR result creating a management dilemma: a case report

**DOI:** 10.1186/s13256-020-02498-3

**Published:** 2020-09-18

**Authors:** Kelli M. Robertson, Christopher L. Harvey, John M. Cunningham

**Affiliations:** 1 Division of Internal Medicine, University of Colorado School of Medicine, 13001 East 17th Place, Aurora, CO 80045 USA; 2 Division of Hospital Medicine, Denver Health Medical Center, 601 Broadway Street, MC 4000, Denver, CO 80203 USA

**Keywords:** Acyclovir, Neurotoxicity, Varicella, Encephalitis, Case report

## Abstract

**Background:**

Varicella zoster virus central nervous system infections can present as aseptic meningitis, encephalitis, myelitis, and vasculopathy. Diagnosis is based on identification of varicella zoster virus deoxyribonucleic acid (DNA) in the cerebrospinal fluid by polymerase chain reaction. Therapy for these infections is acyclovir or valacyclovir. However, acyclovir can have neurotoxic effects that can mimic the presentation of varicella zoster virus central nervous system disease. We present a rare presentation of a patient who had acyclovir-induced neurotoxicity who also had a false-positive cerebrospinal fluid varicella zoster virus polymerase chain reaction result, creating a management dilemma. We review the clinical characteristics of acyclovir-induced neurotoxicity. In addition, we present the diagnostic characteristics of the cerebrospinal fluid viral polymerase chain reaction and alternative methods to diagnose central nervous system varicella zoster virus disease.

**Case presentation:**

A 68-year-old Hispanic man with end-stage renal disease was diagnosed with cutaneous zoster at an outside facility and was started on acyclovir 4 days prior to admission. His family noted worsening confusion, agitation, speech difficulty, and hallucinations, leading them to bring him to the emergency department. His cerebrospinal fluid varicella zoster virus polymerase chain reaction result was positive, indicating the presence of varicella zoster virus deoxyribonucleic acid in the cerebrospinal fluid; however, he did not have cerebrospinal fluid pleocytosis typical of varicella zoster virus meningoencephalitis. Pharmacy records from the outside hospital revealed supratherapeutic acyclovir dosing. This led to a diagnostic dilemma over whether this patient had varicella zoster virus encephalitis or acyclovir-induced neurotoxicity. Acyclovir was discontinued, and the patient underwent two sessions of hemodialysis to remove acyclovir, which led to a full neurologic recovery.

**Conclusions:**

Varicella zoster virus encephalitis and acyclovir-induced neurotoxicity can have similar presentations. Varicella zoster virus deoxyribonucleic acid can be present in the cerebrospinal fluid during active cutaneous zoster in the absence of central nervous system disease. If concern for central nervous system varicella zoster virus disease remains high, additional testing with cerebrospinal fluid serology can be performed. Compared with central nervous system varicella zoster virus disease, acyclovir-induced neurotoxicity has a more predictable clinical resolution once drug therapy is discontinued or the patient undergoes hemodialysis, which can aid in making the diagnosis. Clinicians should be aware of this rare and dangerous complication of acyclovir. In addition, clinicians should have an understanding of the diagnostic limitations of cerebrospinal fluid viral polymerase chain reaction and have alternative approaches available to diagnose central nervous system varicella zoster virus disease when it is suspected.

## Background

Varicella zoster virus (VZV) is one of the more common viral agents to cause central nervous system (CNS) infection [1]. CNS zoster can present as aseptic meningitis, encephalitis, myelitis, and vasculopathy [2–4]. Prior reports suggest that 32–62% of patients have cutaneous manifestations of zoster when neurologic symptoms begin [2, 5]. In patients with VZV encephalitis, mortality rates range from 9% to 20%. Neurologic sequelae, including long-standing cognitive deficits, are common [1, 3, 5]. Due to the high morbidity, current recommendations are for treatment with an antiviral agent as soon as CNS infection is diagnosed [6]. Although VZV meningitis and encephalitis should improve with acyclovir, acyclovir used to treat shingles can also have neurotoxic side effects. Prior case reports have demonstrated that acyclovir-induced neurotoxicity (AIN) presents as an acute encephalopathy, making it difficult to distinguish from VZV encephalitis on the basis of clinical presentation alone [7, 8]. Magnetic resonance imaging (MRI) does not frequently demonstrate disease-specific abnormalities in VZV meningoencephalitis and is unlikely to help differentiate between these two conditions [9]. Cerebrospinal fluid (CSF) analysis can often help differentiate these two conditions, but CSF abnormalities and false-positive PCR results can occur in AIN [7, 8]. Accurately distinguishing these two diagnoses is critical, given their opposing management strategies.

We discuss a rare case of AIN in a patient with end-stage renal disease (ESRD) who presented with a dermatomal rash, acute encephalopathy, and CSF with a positive VZV polymerase chain reaction (PCR) result. We describe the risk factors and clinical features of AIN and review the diagnostic characteristics of viral PCR for VZV. In addition, we discuss an approach for a suspected false-positive PCR result when an urgent decision is needed regarding whether to continue antiviral treatment.

## Case presentation

A 68-year-old Hispanic man with ESRD initially presented to an outside urgent care clinic with a 3-day history of a painful rash over the right thorax. His examination revealed a dermatomal, vesicular eruption at the T9 dermatome consistent with zoster reactivation. He was started on oral acyclovir 800 mg five times daily. The recommended renal dosing for a patient receiving dialysis would have been 200 mg twice daily. Over the following 4 days, his family noted progressively worsening confusion. On the day of admission, the patient’s son called emergency medical services due to the patient’s confusion, agitation, and auditory hallucinations. Prior to this presentation, the patient had lived independently and was able to complete all activities of daily living without assistance. His past medical history included ESRD, hypertension, type 2 diabetes mellitus, anemia due to chronic renal disease, and secondary hyperparathyroidism. Prior to arrival at our hospital, his medications included amlodipine 5 mg oral once daily, aspirin 81 mg oral once daily, metoprolol succinate 150 mg oral once daily, and 20 units of insulin NPH-regular 70–30% twice daily. Oral acyclovir 800 mg five times daily was the only new medication, started 4 days prior to arrival. He received outpatient dialysis three times weekly. Per the report of his family, he did not smoke or drink alcohol. His family was unaware of any family history of neurologic disorders. He was unemployed and had no recent travel exposures. He had not been previously vaccinated against VZV.

**Fig. 1:**
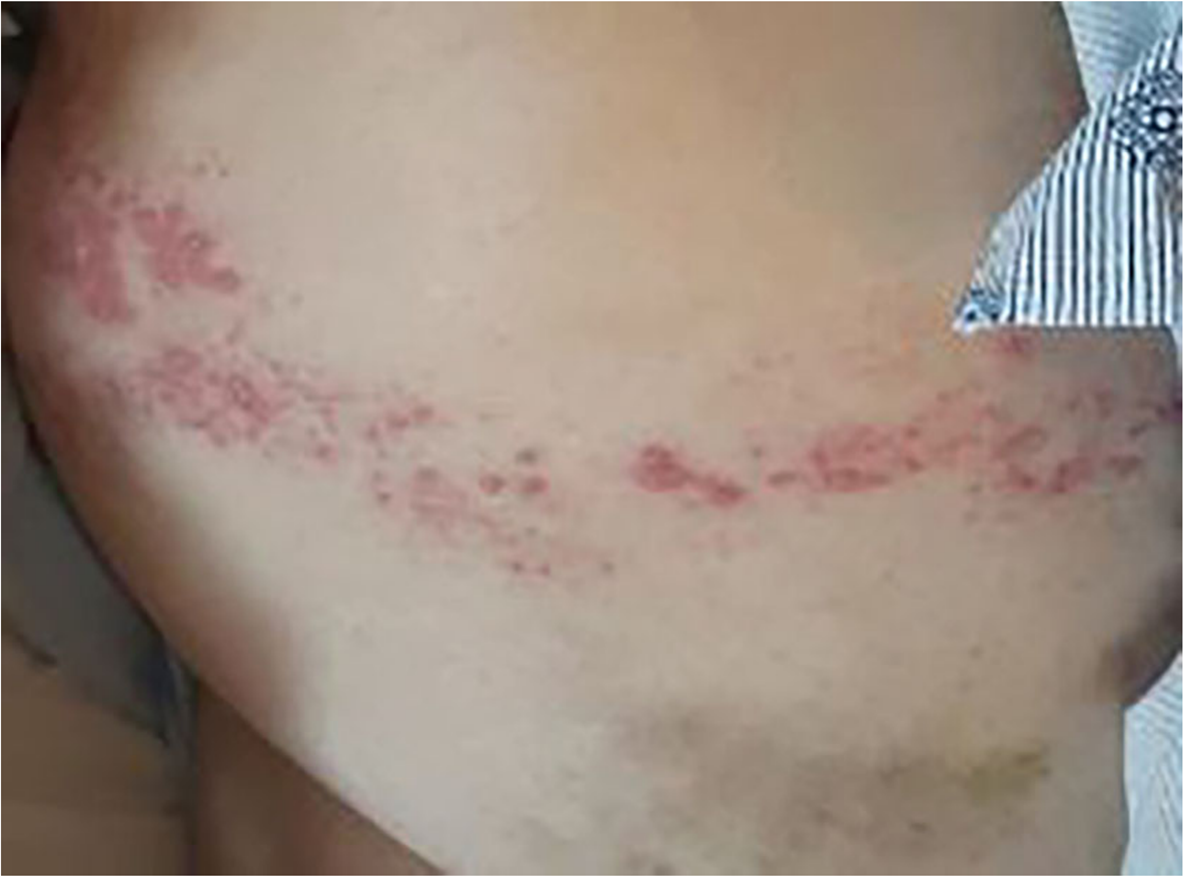
Photograph of a right-sided T9 dermatomal rash present in our patient. The erythematous, papular rash with vesicles in a dermatomal distribution was highly suggestive of zoster reactivation. Other differential diagnoses, including cutaneous candidiasis, impetigo, contact dermatitis, and primary varicella, were less likely based on the patient’s history and the distribution of the rash

Upon physical examination, he was agitated and oriented only to self. His vital signs upon presentation included a body temperature of 37.3 °C, heart rate of 100 beats per minute, blood pressure of 177/90 mmHg, respirations of 20 breaths per minute, and oxygen saturation of 93% on 2 L by nasal cannula. Examination of the patient’s head and neck revealed no signs of trauma. His pupils were equal, round, and reactive to light. His oropharyngeal examination revealed dry mucous membranes but no lesions or ulcers. His pulmonary examination result was clear to auscultation bilaterally. His cardiovascular examination revealed normal heart sounds without murmurs, rubs, or gallops. His abdomen was nontender and without signs of guarding. His extremities were warm, and no lower extremity edema was present. His musculoskeletal examination revealed normal range of motion and no joint erythema or effusions. His skin examination revealed a right-sided rash with erythematous papules with vesicles along the T9 dermatome (see Fig. 1). Upon neurologic examination, he was unable to follow commands for testing cranial nerves, strength, or coordination. His speech was dysarthric but not aphasic. He moved all extremities spontaneously and withdrew to pain. His reflexes were 3+ bilaterally in the upper and lower extremities with no clonus and a negative Babinski sign. His muscle tone was normal.

His laboratory studies were notable for a serum glucose level of 170 mg/dl, sodium level of 133 mEq/L, blood urea nitrogen level of 43 mg/dl, creatinine level of 5.90 mg/dl, calcium level of 8.5 mg/dl, white blood cell count of 6.8 × 10^9^ cells/L, hemoglobin level of 11.5 g/dl, and platelet count of 239 × 10^9^ cells/L. His thyroid-stimulating hormone level was 2.95 mIU/L. The result of his urine drug screen was negative, and his ethanol level was <10 mg/dl. The result of his urinalysis was negative for leukocyte esterase or nitrites.

MRI of the patient’s brain did not reveal large-territory infarct or evidence of vasculopathy. His electroencephalogram demonstrated diffuse slowing and disorganization consistent with a toxic-metabolic encephalopathy, but he had no epileptic discharges. His chest x-ray did not reveal any pulmonary infiltrates or evidence of pneumonia. CSF studies showed a protein level of 85 mg/dl, glucose level of 76 mg/dl, no red blood cells, no white blood cells, and no organisms on Gram staining. His CSF PCR result was positive for VZV and negative for other common etiologies of bacterial or viral encephalitis.

The emergency department did not initially have access to his prior urgent care pharmacy records. Upon receiving the positive VZV CSF PCR result, he was started on intravenous acyclovir renally dosed at 5 mg/kg and was admitted to the medicine service for his acute encephalopathy. With common toxic and metabolic causes of encephalopathy ruled out in the emergency department, our primary differential diagnosis was between VZV encephalitis and AIN. Uremic encephalopathy was also included in our differential diagnosis. However, review of the outpatient dialysis records revealed adequately dosed dialysis, as evident by achieving target dialysis clearance parameters and no missed dialysis sessions, making this diagnosis unlikely. When the outside pharmacy records arrived, revealing inappropriate acyclovir dosing, AIN became the favored diagnosis. Acyclovir was stopped, and serum drug levels were sent to a reference laboratory. CSF titers for anti-VZV immunoglobulin M (IgM) and IgG were also pending at this time.

Nephrology was consulted, and the patient underwent two 4-hour dialysis sessions on consecutive days, after which his neurologic status improved significantly. His hospital course was not complicated by other evidence of infection, electrolyte abnormalities, or hypoglycemia that could have contributed to his acute encephalopathy. By hospital day 4, he had returned to his baseline neurologic status. On hospital day 5, he had a normal neurologic examination result and was discharged to home. At follow-up 6 months later, the patient had experienced no further episodes of confusion, dysarthria, or hallucinations and was living independently. The temporal onset of hallucinations, dysarthria, and confusion after initiating acyclovir therapy combined with the rapid improvement after discontinuation of acyclovir and initiating hemodialysis made AIN the likely diagnosis.

## Discussion

We present a case of a patient with ESRD receiving hemodialysis who presented with the acute onset of encephalopathy, agitation, dysarthria, and hallucinations after starting acyclovir that was inappropriately dosed. His CSF did not reveal a lymphocytic pleocytosis typical of VZV encephalitis; yet, his VZV CSF PCR result was positive. This created a diagnostic dilemma between the competing diagnoses of AIN and VZV encephalitis. The patient’s full neurologic recovery after withholding acyclovir and initiating dialysis was very suggestive of AIN. We suspect the positive CSF VZV PCR result was a false-positive finding. The combination of AIN and a false-positive CSF VZV PCR result is unique in the literature. To make a diagnosis in this case, a clinician must know the clinical features of AIN, understand the CSF VZV PCR’s diagnostic characteristics, and have an approach to a potential false-positive CSF PCR finding. We discuss these issues below.

AIN is a rare but documented consequence of therapy. Most cases occur in the setting of renal impairment and in patients on dialysis [7]. Clinical characteristics (see Table 1) include tremor and myoclonus (58%), confusion (50%), agitation (38%), hallucinations (25%), and dysarthria (17%) [11]. Previous reports suggest that visual hallucinations and dysarthria may be unique to AIN as opposed to VZV encephalitis [8]. Both were present in our patient. Although findings of CSF studies are typically normal, one case of AIN presented with an elevated CSF white blood cell count typical of viral meningoencephalitis but with a negative CSF VZV PCR result [7]. Another case report documented a positive VZV PCR result but with a traumatic tap [8]. Serum levels of acyclovir are often not useful to diagnose AIN, because there can be a substantial delay, with symptoms typically presenting 24 to 48 hours after peak serum acyclovir levels due to the rate of reaching equilibrium between serum and CSF being slow [11]. In addition, the management decision often needs to be made before acyclovir levels become available. The diagnosis is primarily based on the temporal association of neuropsychiatric symptoms and acyclovir administration and clinical improvement after discontinuation of acyclovir. The pathophysiology of AIN is unclear but is speculated to be related to accumulation of the metabolite 9-carboxymethoxymethylguanine (CMMG). Elevation of both serum and CSF levels of CMMG is associated with neuropsychiatric symptoms [10]. CSF CMMG levels were not obtained in our patient’s case, because this test was unavailable at our institution. Prior reports show that discontinuation of acyclovir leads to improvement in neurologic symptoms over 48–72 hours and complete recovery over 5 days [7]. Patients with ESRD who undergo extended hemodialysis may have a faster recovery because 60% of acyclovir is removed in a 6-hour dialysis session, which may aid in the diagnosis of AIN [7, 8, 12]. In our patient with ESRD, the presentation with acute encephalopathy in the setting of incorrectly dosed acyclovir was highly consistent with AIN. However, consideration of the impact of the initial positive CSF VZV PCR result was an important factor in managing this case.

**Table 1:** Clinical characteristics of varicella zoster virus encephalitis compared with acyclovir-induced neurotoxicity

	VZV encephalitis	Acyclovir-induced neurotoxicity
Timing of onset	• Variable temporal association with cutaneous zoster and primary infection	• Close temporal association with initiation of acyclovir
Risk factors	• Immunocompromised state	• Impaired renal function
	• Can occur in previously health individuals	• Incorrect dosing
Clinical characteristics^a^	• Headache	• Acute encephalopathy
	• Acute encephalopathy	• Tremor, myoclonus
	• Fever	• Agitation
	• Nausea and vomiting	• Hallucinations
	• Cutaneous zoster may be present	• Dysarthria
CSF studies	• CSF pleocytosis	• Often normal
	• Positive CSF VZV PCR	• May have CSF pleocytosis [7] or positive VZV PCR [8]
	• Positive CSF anti-VZV IgM or IgG^b^	
	• Elevated serum or CSF CMMG^c^	
Treatment	• Acyclovir or valacyclovir	• Cessation of acyclovir
		• Hemodialysis
Clinical course	• Variable resolution	• Full resolution over 1–5 days
	• Chronic neurologic sequelae may occur	

*Abbreviations: CMMG* 9-Carboxymethoxymethylguanine, *CSF* Cerebrospinal fluid, *Ig* Immunoglobulin, *PCR* Polymerase chain reaction, *VZV* Varicella zoster virus

^a^These are more classic presentations. There is considerable clinical overlap in these two diagnoses.

^b^Positive CSF anti-VZV IgM and IgG may take several days or weeks to develop and would require repeat lumbar puncture.

^c^Elevated serum and CSF CMMG levels are suggestive of acyclovir-induced neurotoxicity [10].

Molecular diagnostic assays for common agents causing meningitis and encephalitis are sensitive, specific, and rapid; however, false-positive results are a concern [13]. A prior study evaluating the diagnostic characteristics of PCR for common viral CNS infections using virus-specific case definitions found that up to 22% of positive findings for VZV were possible false-positive results. This was either due to not meeting the criteria for encephalitis or an alternative diagnosis being probable [14]. In one study validating a combined meningitis/encephalitis CSF panel, one of the seven positive samples for VZV was a false-positive result [13].

There are several possibilities for a positive CSF VZV PCR finding in the absence of CNS VZV disease. First, if there is circulating VZV DNA in the serum during zoster reactivation, a traumatic tap could lead to a positive CSF sample. Contamination of the laboratory specimen would be a second cause. Looking at the CSF red blood cell count and considering a repeat lumbar puncture addresses these two causes. Third, VZV DNA may have gained access to the CSF during reactivation in the dorsal root ganglia, but its presence was not indicative of active CNS zoster infection. Supporting this possibility is a prior study which found that 10 (21.7%) of 46 CSF samples from patients with active cutaneous zoster but without CNS manifestations had a positive VZV CSF PCR result [15]. This is the most likely explanation in our patient’s case, given the absence of an inflammatory response in the CSF or evidence of a traumatic tap.

The final scenario to consider in our patient, given the positive CSF VZV PCR finding, was that a CNS zoster infection was present but that he also had AIN. Although most patients with VZV meningoencephalitis have an abnormal CSF white blood cell count, it can be absent in VZV vasculopathy [4]. Although CSF pleocytosis may be absent in VZV vasculopathy, imaging abnormalities are present in 97% of cases [4]. In addition, the detection of intrathecal anti-VZV IgG is specific for CNS VZV disease and VZV vasculopathy [4, 16–18]. Our patient had normal imaging findings and negative VZV CSF serologies, making this scenario less likely. CSF anti-VZV IgM and IgG take several days to rise after VZV infection, but they will remain positive in the CSF for months, whereas VZV DNA would likely clear from the CSF over the course of weeks [3]. As a result, serology’s role may be greater in cases in which CNS VZV disease is suspected but the rash is absent and the CSF PCR result is negative [16]. Although serologies are helpful in identifying CNS VZV disease, they may take several days to return. If VZV or HSV encephalitis is suspected and there is also concern for AIN, consultation with an infectious disease specialist and a pharmacist can help determine when acyclovir treatment should be restarted and the appropriate acyclovir dose.

## Case conclusion and summary

After discontinuation of acyclovir and two hemodialysis sessions on consecutive days, our patient had significant clinical improvement. On hospital day 5, his anti-VZV IgM and IgG levels from the CSF were negative. His acyclovir levels were elevated at 3.7 μg/ml (three times the upper limit of normal for a trough value), further supporting a diagnosis of AIN. Because complete clinical recovery occurred while withholding acyclovir, repeat lumbar puncture to repeat anti-VZV titers or the viral PCR was not performed. During a follow-up phone call with the patient and his family 6 months later, the patient had experienced no neurologic sequelae and had no presentations to other facilities for neurologic symptoms.

In summary, AIN is a rare dose-dependent side effect of acyclovir therapy. It should be considered in the differential diagnosis when patients receiving acyclovir present with acute encephalopathy. It most often occurs in the setting of impaired renal function. The clinical presentation of AIN has significant overlap with the clinical presentation of VZV encephalitis [7]. Positive molecular testing of VZV in the CSF may occur in the setting of acute cutaneous zoster in the absence of actual CNS VZV disease [15]. False-positive results with the molecular PCR panel have been documented as well [13]. Clinicians will face the challenging decision of whether to continue acyclovir therapy in this setting. Results of additional testing of the CSF for anti-VZV antibodies and acyclovir levels may take several days to return. Compared with VZV encephalitis, AIN has a characteristic pattern of clinical resolution that may retrospectively aid in diagnosis. Patients with AIN typically have a complete resolution within 1–5 days following acyclovir discontinuation, depending on if the patient undergoes dialysis [8, 12]. We based our decision to discontinue acyclovir therapy on our patient’s normal CSF white blood cell count, history of incorrect acyclovir dosing, and rapid clinical improvement after the first session of dialysis. Ensuring appropriate clinical care requires careful consideration of alternative etiologies for altered mental status, knowledge of this rare side effect of acyclovir, and, finally, knowledge of what additional testing can either confirm or rule out active VZV CNS disease.

## Data Availability

All necessary data and material are provided.

## Abbreviations

AIN: Acyclovir-induced neurotoxicity
CMMG: 9-Carboxymethoxymethylguanine
CNS: Central nervous system
CSF: Cerebrospinal fluid
ESRD: End-stage renal disease
Ig: Immunoglobulin
MRI: Magnetic resonance imaging
PCR: Polymerase chain reaction
VZV: Varicella zoster virus
